# Patient Experiences of Seeking Specialized Mental Health Care in Norway: A Qualitative Study

**DOI:** 10.1177/21501319251350601

**Published:** 2025-07-09

**Authors:** Silje Sørum, Veronica Lockertsen, Sara Rivenes Lafontan

**Affiliations:** 1 OsloMet*—*Oslo Metropolitan University, Norway

**Keywords:** care pathways, mental health services, mental help-seeking, primary health care, general practice, patient perspective

## Abstract

**Background:**

Despite the increasing prevalence of mental health concerns in Norway, limited knowledge exists regarding how patients experience accessing specialized mental health services through primary care. General Practitioners (GPs) act as crucial gatekeepers, yet their interactions and referral practices significantly influence patients’ help-seeking trajectories. This study aims to explore patient perspectives on the role of GPs as gatekeepers to specialized mental health care, with a focus on communication, decision-making, and navigation challenges within the Norwegian healthcare system.

**Methods:**

An exploratory qualitative study was conducted involving semi-structured interviews with 13 adults (9 women and 4 men), aged 20 to 43 years, who had been referred to specialized mental healthcare in the past 2 years. Participants were recruited using a pragmatic, self-recruitment strategy via social media platforms, and community mental health services. Interviews were conducted between September and October 2021, transcribed verbatim, and analyzed thematically using Braun and Clarke’s 6-step reflexive thematic analysis.

**Results:**

Three main themes were generated: (1) “The critical role of GP acknowledgment”: participants felt deeply affected by whether their GP validated or dismissed their emotional distress; (2) “An arbitrary choice of treatment”: patients reported confusion due to insufficient guidance from GPs, inadequate available information, and perceived randomness in referral practices; and (3) “A degrading process”: participants described feeling powerless, frustrated, and personally responsible for their difficulties navigating the referral system, often exacerbating their mental health symptoms.

**Conclusion:**

Patients frequently experienced feeling unsupported and overwhelmed due to poor GP engagement, inadequate information, and complex referral procedures. Enhancing GP mental health literacy, providing decision-making aids, and creating a centralized digital referral platform could significantly improve patient experiences, reduce waiting times, and foster a more effective, equitable, and patient-centered mental health care system.

## Introduction

Almost a billion people live with mental disorders worldwide, yet accessible mental health care remains scarce.^1,2^ In 2020, approximately 15% of men and 22% of women in Norway consulted primary care services for mental health concerns.^3^ Depression, sleep disorders, stress and anxiety, memory problems, and substance abuse are among the most common mental health challenges encountered by general practitioners.^4^

Recognizing the risks of untreated mental disorders, the Norwegian Ministry of Health endorses early intervention to prevent complex or treatment-resistant conditions.^5^ If left unaddressed mental health issues can affect individuals’ long-term functioning and generate significant societal costs from increased healthcare use, unemployment, and antisocial behaviors.^3^

Management of mental health disorders often combines psychotherapy and medication, depending on severity and patient preference.^6^ Norwegian guidelines recommend a stepped-care approach, whereby treatment starts at the lowest effective level, with GPs assessing whether specialized referral is necessary.^5,7^ If primary care proves insufficient, patients move to higher tiers of care. Norway’s public healthcare system comprises 3 tiers: (1) primary services (GPs, school nurses, and low-threshold community options), (2) specialized mental health facilities (Child and Adolescent Mental Health Services and Adult Mental Health Services), and (3) regional centers for specific conditions (eg, eating disorders). Norway’s health system is comparatively well staffed and funded, yet demand for services still outpaces its capacity. In line with other Western countries, the number of in-patient beds in Norway has been reduced by half over the past two decades, and community-based care is the prevalent ideology.^8^ A longstanding GP shortage, combined with time constraints and bureaucratic fragmentation, places considerable strain on practitioners tasked with referral and follow-up. One strategy to empower patients and streamline treatment pathways, is through shared decision-making (SDM).^9^ A commonly used SDM model consists of 3 elements: (1) The GP recognizes that the patient has multiple options and offers support in exploring them; (2) delivers clear information about each option; and (3) encourages the patient to share their preferences, enabling a joint decision on the most suitable treatment.^9^ SDM requires health literacy, defined by WHO as the ability “gain access to, understand and use information in ways which promote and maintain good health.”^10^ One tool to support SDM, are Helsenorge. Helsenorge is a national e-health portal—serves both patients and GPs. While patients can self-book visits, renew prescriptions, message the clinic, and track referrals, GP’s can use the same system to process e-consultations, prescriptions, and electronic referrals, cutting phone traffic, and improving records. Because specialist mental health care is usually accessed through a GP referral, clear oversight of available qualified services is essential. Although HelseNorge provides a list of registered therapists, there is no unified, regularly updated database encompassing all tiers of mental health treatment.^11^ As the available information about treatment is poorly adapted to people struggling with mental health issues, navigating the pathways between services is challenging. In MacDonald et al^12^ ’s review of young people’s experiences initiating contact with health care services, they identified that service response-related themes included complex pathways, waitlists, eligibility, and fragmented care. They also identified mental health literacy as one key aspect, as both youth and carers reported not realizing that services existed or had knowledge about how to assess their mental health issues.^13^ Although patients would like their GP to be more involved in decisions about treatment options before sending the referral, studies have found that patients received limited support from the GP to decide which treatment option to be referred to. This lack of support is perceived as particularly difficult for patients suffering from depression.^14-16^

In Norway, only 20% of patients with a new diagnosis of depression were referred to specialized care. Meanwhile, patients who can afford private care may circumvent waitlists, creating additional complexity as private psychologists or psychiatrists can either operate within the public system or accept direct payment, and considerable variation exists in waiting times and admission procedures across regions.^17^ Several studies have highlighted that, despite variations in admission procedures, there is a strong correlation between the level of self-reported mental distress and the likelihood of receiving a psychological diagnosis from a GP.^15,16^

Research indicates that patients generally wish to be involved in decision-making regarding their mental health treatment.^18^ However, many report insufficient information, long waitlists, and fragmented care pathways,^19-22^ making the system difficult to navigate.^10^ The GP, serving as gatekeeper, is pivotal in providing initial support, guiding patients through referrals, and shaping their early treatment experiences. Yet GPs themselves face increasing pressures and professional disempowerment, potentially limiting their ability to offer thorough, patient-centered follow-up.

Despite the importance of GP-mediated referrals, there is limited knowledge on how Norwegian adults perceive the process of seeking specialized mental health services beyond primary care—specifically, how they view GP interactions during this journey. Understanding these experiences is crucial for identifying system shortcomings and improving care pathways, communication, and resource allocation.

This study aims to explore patient perspectives on the role of general practitioners as gatekeepers to specialized mental health care, focusing on communication, decision-making, and navigation challenges in the Norwegian healthcare system.

## Methods

We selected a qualitative, exploratory design as the study aims to explore patient perspectives on the role of GPs as gatekeepers to specialized mental health care. This objective calls for rich, first-person accounts of emotions, perceptions, and contextual factors. A qualitative approach was considered suitable to address the study aim.^23,24^ We drew upon the conceptualization of health literacy to explore participants’ capacity to obtain, process, and understand basic health information and services relevant to their mental health referrals. The criteria for reporting qualitative research (COREQ) 32-item checklist served as a reference for reporting the present study.^25^

### Recruitment and Participants

The inclusion criteria were that the participants were between 18 and 45 years old, spoke Norwegian and had been referred to specialized mental health care treatment during the past 2 years. Information about the referral diagnosis was not collected. A pragmatic, self-recruitment strategy was employed to recruit participants; information about the study and a request for participation was shared on the 2 Norwegian Instagram accounts named “Psyktærlig” and “Angsthumor.” In addition, the same information was posted at a rehabilitation center for people with mental health problems, “Fontenehuset,” in Oslo. People who expressed interest in participating in the study received additional written information about the study by email before consenting to participate. The study employed a convenience sampling approach; however, efforts were made to achieve variation in the sample to ensure data richness.^24^ The final sample comprised 9 women and 4 men (see Table 2) drawn from all 5 major geographical regions of Norway. Most participants had been referred to mental health treatment multiple times over the past 2 years.

### Data Collection and Reflexivity

The semi-structured interviews were conducted by the first author from September to October 2021. The interviews lasted on average 1 h, 17 min. The interview guide (Supplemental File 1) consisted of open-ended questions about the experience of seeking specialized mental healthcare beyond primary healthcare and included questions about health literacy, such as “Can you tell me how you went about seeking information about the different mental health treatment options?” and “Can you tell me about the information you received from your GP about the different treatment options?” As shared decision-making informed our investigation of participants’ perceived involvement in decisions about referral destinations and treatment plans, participants were asked whether, and in what ways, their GP included them in these decisions.

The interview guide was pilot tested and minor revisions to question phrasing were made to improve clarity. Due to COVID-19 restrictions and the geographical spread of the participants, the interviews were carried out using the videotelephony software program Zoom (12 interviews) and face to face (1 interview) depending on the participants’ preference. They were recorded and transcribed verbatim and de-identified by the first author using participant codes.

Throughout the conceptualization, data collection, and analysis, the authors regularly discussed preconceptions about GP-patient relationships in Norwegian healthcare. We entered the study with a pre-understanding that health literacy and shared decision-making were key concepts in this context. Initially implicit, these concepts later became explicit and served as the theoretical framework guiding the study’s conceptualization and focus.^26^ Both concepts were relevant for understanding how patients acquire information, interpret treatment options, and engage in the decision-making process with healthcare professionals. Memos and reflexive notes were maintained by the first author to ensure transparency regarding evolving interpretations of the data.^27^ The 3 authors have academic and professional backgrounds in nursing, mental health care and early childhood development.

### Data Analysis

The data material was analyzed using Braun and Clarke’s 6-step reflexive thematic analysis in order to identify patterns and themes relevant to the study aim.^28^ The 6 steps are: (1) Familiarization with the data. (2) Generating initial codes. (3) Generating themes. (4) Reviewing themes. (5) Defining and naming themes. (6) Producing the report. The analytical process was carried out in a flexible way where we moved back and forth between the different steps. In the first step, the first author listened to the recorded interviews, and transcribed the data and all authors read and re-read the transcribed data and wrote down first impressions of the data, which was later discussed. In the second step, the first author developed codes which were discussed with the last author. Here, the theoretical framework guided the coding of interview transcripts for references to patient autonomy, information-sharing, and mutual decision-making. In the following step, the first and last author determined which codes fit together and constructed initial themes. In the fourth step, initial themes were discussed among all authors and the themes were modified to avoid overlaps. In the last step of the analytic process, before producing the report in the form of the present manuscript, we finalized the themes with emphasis on developing themes that were meaning-based interpretive stories and ensured that they responded to the study aim.^29^ The data analysis was primarily inductive; however, the concepts of health literacy and shared decision-making provided a guiding framework to ensure that the final themes captured both the participants’ experiences and the underlying processes of communication, empowerment, and decision-making central to mental health help-seeking. Table 1 below provides an illustration of the analytical process.

**Table 1.** Illustration of the Analytical Process.

Data extract	Codes	Theme
“(. . .) the GP shifted that burden onto me—I had to track down the information on my own, in a jungle of information.”	General practitioner (GP) Negative Lack of shared decision-making	An arbitrary choice of treatment

## Results

Three themes were identified: the critical role of GP acknowledgment, an arbitrary choice of treatment, and a degrading process. Demographic characteristics of the participants are described in Table 2.

**Table 2.** Characteristics of the Participants.

Participant	Gender and age	Employed/studying (y/n)
1.	Female, age 20 years	Y
2.	Female, age 32 years	Y
3.	Female, age 43 years	Y (sick leave)
4.	Female, age 31 years	Y (sick leave)
5.	Female, age 32 years	No data
6.	Male, age 34 years	N
7.	Female, age 37 years	Y (sick leave)
8.	Female, age 23 years	No data
9.	Female, age 31 years	Y
10.	Female, age 33 years	Y
11.	Male, age 28 years	Y
12.	Male, age 32 years	Y (sick leave)
13.	Male, age 32 years	No data

### The Critical Role of GP Acknowledgment

In the present study, many of the participants reported feeling neglected and unsupported by the GP during their journey to seek specialized mental healthcare. They described a feeling that the GP did not relate or acknowledge their level of suffering and distress. This contributed to a sense of being overwhelmed by the responsibility of identifying suitable mental health treatment options. The participants who experienced support from their GP felt that the GP understood their situation and emotional state. As a result, they experienced an emotional relief and felt greatly helped by the GP. Having a GP who understood them and their mental health condition, and who created a space for open communication, made the participants feel supported and hopeful (quote 1, Table 3). Conversely, participants who felt unsupported by their GP felt dismissed and believed the GP was uninterested in them. This perception arose when GPs skipped follow-up questions or when participants sensed that the doctor doubted whether they were “ill enough.” As a result, participants found it difficult to be open about how they truly felt and what their mental health issues were. For some, the natural response was to hold back, as they did not allow themselves to that degree of vulnerability. Others responded more emotionally to the situation, thereby revealing the severity of their distress to the GP. Some experienced that this led to them being met with better support from the GP (quote 2, Table 3).

**Table 3.** Verbatim Quotes Per Theme.

The critical role of GP acknowledgment	
Quote number	
1	*It is a process that becomes very difficult because you don’t know where to start, and I was in a pretty bad place in general, so it was very difficult to know where to start at all. I completely lost hope, but my GP sort of stood up for me and said, “we’re going to get this done”. So, she followed me up until I got an appointment specialist. I was allowed to come to her once a week until specialized mental health treatment was available. She was not specialized in mental health, but she was empathetic, and I felt important to her. So that was enough in a way.* (Participant 8)
2	*If it hadn’t been for that consultation with the GP going quite badly, I don’t think I would have gotten the help I needed. The GP only understood that it was serious because I had the reactions I had during the consultation. If I had just sat there and said that this is important, without having any reactions, I don’t think I would have gotten that help*. (Participant 10)
An arbitrary choice of treatment	
3	*I remember I got a bit annoyed with him like, “give me a name, say a place, something!” So, then he just googles, and just like that, “yeees, there’s probably this one (. . .), also there’s one there” and just sort of “so, do you want the number or not?”* (Participant7).
4	*So, you’re like some kid in the supermarket and just like, “who’s there?” a bit like that. Very, very lost*. (Participant 2)
5	*Yes, now it’s just a matter of shooting blindly and hoping it turns out right* (Participant 8).
6	*Because I don’t have the opportunity to drive for 3 hours, once a week. (. . .) and miss work* (Participant1).
A degrading process	
8	*You already feel like a failure, and then you feel like you’re not even capable of having a dialogue with your GP. He’s not even interested in you. So, then I felt that I’m not interesting enough, I’m not good enough at explaining how I feel* (Participant 3).
9	*I am left with little hope, and courage and motivation (. . .) yes, an overall negative impression of the whole process. . . from the second you go to the GP until you have had your first appointment with a therapist* (Participant 1).
10	*I’m going to have to give up my job for sure. Because I can’t go back to the job I have. I wish I had had a GP who had been a bit more proactive, so maybe I could have been half a year ahead of how I am doing now or. . . be in a better place than I am now. I sort of feel like I’ve spent a year of my life just lying in bed in a dark room and not really getting much help.* (Participant 5)

### An Arbitrary Choice of Treatment

The participants believed that it was not only difficult for them to find the right treatment option, but that the GPs also did not have a good overview of where they could refer them. They found it difficult that the GPs often had limited knowledge of which treatment options were suitable and which therapists were available. This was particularly a problem for those participants who did not have a preference about future treatment but instead wanted the GP to decide for them (quote 3, Table 3). In searching for suitable treatment, both the participants and their GP were reliant on the quality of available information on Helsenorge and the internet. The participants experienced abundant information about different types of treatment, but minimal information about each individual treatment option, waiting times, and the application process, which made it difficult to sort out what was relevant to them. In their search for the appropriate treatment, participants felt overwhelmed by the numerous options available with no one to guide them (quote 4, Table 3). For the participants, it could be difficult to understand why they had to go through the GP, as the referral process often felt arbitrary (quote 5, Table 3). After being referred by their GP, many participants felt “thrown around” within a confusing and complex system, where they themselves had to follow up on referrals and contact relevant treatment facilities to obtain information. They also experienced that there were significant regional differences in treatment options in Norway, which posed a challenge for those living far from what seemed like a suitable treatment option. Those participants were dependent on finding a treatment option that enabled them to maintain everyday life and work. The participants experienced the urbanization of treatment options as a major impediment to their ability to receive the treatment they believed was best for them. Instead, they had to accept what was available near where they lived (quote 6, Table 3).

### A Degrading Process

When the participants described the process of finding a suitable treatment option in collaboration with the GP, they talked about how they had feelings like hopelessness, anger, frustration, powerlessness, and not being good enough. Many placed the blame and responsibility on themselves, believing that they had not been good enough in communicating their complaints to the GP (quote 8, Table 3). They acknowledged that their way of communication influenced how the GP were able to help them. But they found it difficult to explain and find the right words and expressions, as they “*were not a psychologist or had sufficient knowledge about mental health*.” Many also described feeling unworthy and stupid because they could not find information about suitable mental health treatment. The participants believed they paid a heavy price for the time it took from referral until they received specialized mental health treatment. Some described experiencing a worsening of their symptoms and that their mental health deteriorated during this period (quotes 9 and 10, Table 3).

## Discussion

In the present study, participants reported feeling neglected and unsupported by their GP during their journey to seek specialized mental healthcare. They described a sense of being overwhelmed with the responsibility of identifying suitable mental health treatment options due to insufficient guidance. The treatment referral process was perceived as arbitrary, characterized by a lack of information, and regional disparities. From the perspective of health literacy and shared decision making, the study findings underscore the structural and relational barriers that can exacerbate patient distress.

In our study, many participants described feeling burdened by the responsibility of identifying appropriate treatment options on their own. This self-directed search for suitable care may be viewed as part of the shared decision-making (SDM) process and can be interpreted as a strategy that promotes active patient involvement in treatment. SDM, grounded in the principles of patient-centered care, is a collaborative process in which clinicians and patients work together to select treatment options that align with both the patient’s preferences and the best available clinical evidence.^9,30,31^ However, as in other studies, the participants experienced this responsibility as overwhelming. In some cases, it intensified their symptoms and undermined the dignity-preserving promise of SDM.^32^ Although most of the participants in our study were working and may have been perceived by the GP as highly functional, they still reported limited knowledge of the health system and found the process difficult to manage. Patients often want to exercise autonomy, while also having their limitations and need for help acknowledged, that way SDM can be empowering rather than overwhelming.^33^

Many participants in the present study found communication with their GP difficult. Previous studies have also found that patients with mental health disorders often struggle to communicate symptoms to their GP.^20^ Effective communication between the patient and their GP is essential for the patient to feel supported and to find the best treatment option.^9^ The study findings underscore the importance of establishing trust within the doctor–patient relationship, as well as the need for GPs to thoroughly assess patients’ personal resources and mental health literacy when engaging in shared decision-making. Not all patients wish to be equally involved in decisions or assume shared responsibility for health choices.^18,22^ Indeed, some participants in our study wanted the GP to decide on the best treatment option, believing the GP knew and understood them well enough to make the right decision. This reinforces the current thinking that shared decision-making requires open, trust-based clinician-patient dialogue.^18^ In addition, many patients seeking help for mental health problems have symptoms that negatively affect their self-esteem, sense of self-worth, and worthiness to receive care.^34^ Thus, patients with mental health problems are particularly vulnerable to their GP’s approach. Furthermore, previous studies have found that GPs often overestimate their own communication skills, as well as the mental health literacy of their patients.^35^ As the gatekeeper to mental health treatment, it is essential that the GP is adequately trained to recognize and diagnose mental health problems, given that patients often cannot verbalize their experienced suffering and deteriorated function.^12,36^

Previous studies have found that doctors believe they carry out shared decision-making, whereas patients feel less empowered and less involved in treatment choices.^37,38^ The study findings highlight a critical tension in how SDM is understood and implemented in clinical practice which highlights, emphasizing the importance of not treating SDM as an uncontested concept. While SDM is commonly defined as a collaborative process, it risks becoming a form of “symbolic liberalism.” In such cases, patients are presented with choices without receiving adequate relational support, mutual understanding, or meaningful guidance from their GP. These findings emphasize the importance of balancing patient autonomy with genuine relational support. SDM should not be reduced to a superficial exercise in offering choices, but rather serve to uphold patient dignity without reverting to paternalistic care. Although GP’s are expected to provide mental-health care through a SDM approach, the emphasis must be on creating a mutual understanding through dialogue of information and create an agreement and understanding of each other’s preferences.^32^ In addition, when the GPs lack an overview of suitable for the patients,^39^ it is questionable why the patients—already low on resources and system knowledge—are left to navigate the help-seeking process alone.^39^

Many patients are often reluctant to seek help for mental problems, *as they wish to manage on their own and perceive needing help as shameful*.^20^ Only 26% of people with mental disorders are estimated to seek health care. Those who do seek help often delay contacting their GP in an attempt to manage on their own, which indicates that patients may experience a societal or personal threshold in seeking professional help for mental health problems.^7^ With barriers such as mental health stigma and difficulties accessing help, patients usually have suffered a long time even before entering the health care system.^20^ There is an ongoing debate about the impact of the GP’s gatekeeping role regarding healthcare utilization and patient safety.^40,41^ Our study’s findings largely confirm the gatekeeper role of the GP described in the literature.^40^ Many GPs find identifying with and filling the gatekeeper role difficult, feeling caught between being both gatekeepers and patient advocates.^42,43^ In Norway, GPs report high workload and work-related stress, contributing to professional disempowerment.^44,45^ There are concerns that Norway’s GP system may collapse unless measures are implemented, due to the unsustainable workload currently placed on GPs.^34^ Thus, while GPs are often aware of their patients’ disempowered position, structural challenges may limit how fully they can meet patients’ needs.^46^ Our findings indicate that enhancing GPs’ capabilities and resources is key to ensuring that patients feel supported and maintain dignity throughout the mental health help-seeking process. Specific interventions such as implementing a centralized digital referral platform, enhancing GP education in mental health literacy, and developing decision aids to facilitate shared decision-making could help reduce patient burden and streamline care pathways.

### Study Strengths and Limitations

One limitation of the study could be that the participants were primarily recruited using sites on Instagram targeting people struggling with mental issues. This may have excluded individuals with limited digital access, lower health literacy, or deeper distrust in healthcare services—can be under-represented. While these channels enabled access to information-rich participants, it may create a bias towards attracting more vocal or dissatisfied individuals, leading to the recruitment of individuals with particularly negative experiences regarding GP support. The study does not include people who, for various reasons, were not referred to mental health treatment or who dropped out of the process; whose perspectives might have yielded richer data. Most participants were working, which might have influenced their help-seeking strategies and how their GPs perceived them. Moreover, data were not collected about participants’ specific mental health symptoms or diagnoses, as the quality of GP interactions and referral complexities are often shared concerns across various mental health conditions. Nevertheless, the lack of diagnostic data and the fact that several of the participants had been referred to specialist services several times constrains how finely one can interpret the findings. Because we did not systematically capture these variations, the analysis may underrepresent how different conditions intersect with the structural barriers we highlight in the study. Including such details in future research could help contextualize findings further. Aiming for “information power,” the sample size was considered adequate to allow for transferability to other contexts and appropriate to answer the study aim.^29^

## Conclusions

Overall, these findings highlight how patients seeking specialized mental healthcare often felt unsupported and overwhelmed, particularly when the responsibility for identifying appropriate treatment options largely fell on them. While GPs play a crucial gatekeeping role, communication barriers, workload pressures, and structural hurdles can undermine the quality of care.

Although the findings cannot be broadly generalized, they provide valuable insights into critical areas that require attention within primary care practice. Ensuring effective SDM requires more than simply presenting choices; it demands careful to consideration of patients’ mental health literacy, emotional needs, and individual preferences for involvement.

Targeted interventions, such as centralized digital referral platforms, enhanced GP training in mental health literacy, and the integration of decision aids into clinical practice, could help address structural gaps in the referral system, strengthen patient support, and promote more equitable care. By enhancing these resources, patients may feel more empowered, while GPs can provide care that supports patients’ dignity and well-being throughout the mental health help-seeking process.

## Supplemental Material

sj-pdf-1-jpc-10.1177_21501319251350601 – Supplemental material for Patient Experiences of Seeking Specialized Mental Health Care in Norway: A Qualitative Study Supplemental material, sj-pdf-1-jpc-10.1177_21501319251350601 for Patient Experiences of Seeking Specialized Mental Health Care in Norway: A Qualitative Study by Silje Sørum, Veronica Lockertsen and Sara Rivenes Lafontan in Journal of Primary Care & Community Health
