# Supplementary material for: Patient Experiences of Seeking Specialized Mental Health Care in Norway: A Qualitative Study
Source: J Prim Care Community Health. 2025 Jul 9;16:21501319251350601. doi: 10.1177/21501319251350601 (PMC12254660; doi:10.1177/21501319251350601)
Supplement: sj-pdf-1-jpc-10.1177_21501319251350601 – Supplemental material for Patient Experiences of Seeking Specialized Mental Health Care in Norway: A Qualitative Study [file sj-pdf-1-jpc-10.1177_21501319251350601.pdf]

Interview guide- experiences of seeking specialized mental health care in Norway

How old are you?

▪ In which part of the country do you live?

▪ Are you a woman or a man?

▪ When did you seek treatment for mental health care complaints?

- Can you tell us about how you went about finding information about mental health care? (internet, friends/family and GP)

- Can you tell us about what worked in this process so that you got information?
- Can you tell what was experienced as challenging/difficult?

- Do you have any thoughts on what could make it easier to find information about mental health care?

- What information do you wish you had before you sought mental health care?
